# Evaluation of the Cherokee Nation Hepatitis C Virus Elimination Program in the First 22 Months of Implementation

**DOI:** 10.1001/jamanetworkopen.2020.30427

**Published:** 2020-12-18

**Authors:** Jorge Mera, Mary B. Williams, Whitney Essex, Kaitlin M. McGrew, Lindsay Boeckman, David Gahn, Anna Miller, David Durham, Jan Fox, Crystal David, Tara Ritter, Stephen Jones, Sally Bouse, Alison Galvani, John W. Ward, Douglas A. Drevets, Hélène Carabin

**Affiliations:** 1 Cherokee Nation Health Services, Tahlequah, Oklahoma; 2 Department of Biostatistics and Epidemiology, Hudson College of Public Health, University of Oklahoma Health Sciences Center, Oklahoma City; 3 Department of Family and Community Medicine, School of Community Medicine, University of Oklahoma Health Sciences Center, Tulsa; 4 Center for Infectious Diseases Modeling and Analysis, Yale School of Public Health, New Haven, Connecticut; 5 Oklahoma State Department of Health, Oklahoma City; 6 Oklahoma State University Center for Health Sciences, Tulsa; 7 Coalition for Global Hepatitis Elimination, The Task Force for Global Health, Decatur, Georgia; 8 Hubert Department of Global Health, Rollins School of Public Health, Emory University, Atlanta, Georgia; 9 Section of Infectious Diseases, Department of Internal Medicine, University of Oklahoma Health Sciences Center, Oklahoma City; 10 Medical Services, Department of Veterans Affairs Medical Center, Oklahoma City, Oklahoma; 11 Département de Pathologie et Microbiologie, Université de Montréal, Montréal, Quebec, Canada; 12 Département de Médecine Sociale et Préventive, Université de Montréal, Montréal, Quebec, Canada; 13 Centre de Recherche en Santé Publique, Montréal, Quebec, Canada

## Abstract

**Importance:**

In 2019, hepatitis C virus (HCV) infection contributed to more deaths in the US than 60 other notifiable infectious diseases combined. The incidence of and mortality associated with HCV infection are highest among American Indian and Alaska Native individuals.

**Objective:**

To evaluate the association of the Cherokee Nation (CN) HCV elimination program with each element of the cascade of care: HCV screening, linkage to care, treatment, and cure.

**Design, Setting, and Participants:**

This cohort study used data from the CN Health Services (CNHS), which serves approximately 132 000 American Indian and Alaska Native individuals residing in the 14-county CN reservation in rural northeastern Oklahoma. Data from the first 22 months of implementation (November 1, 2015, to August 31, 2017) of an HCV elimination program were compared with those from the pre–elimination program period (October 1, 2012, to October 31, 2015). The analysis included American Indian and Alaska Native individuals aged 20 to 69 years who accessed care through the CNHS between October 1, 2012, and August 31, 2017. Cure data were recorded through April 15, 2018.

**Exposure:**

The CN HCV elimination program.

**Main Outcomes and Measures:**

The main outcomes were the proportions of the population screened for HCV, diagnosed with current HCV infection, linked to care, treated, and cured during the initial 22 months of the elimination program period and the pre–elimination program period. Data from electronic health records and an HCV treatment database were analyzed. The cumulative incidence of HCV infection in this population was estimated using bayesian analyses.

**Results:**

Among the 74 039 eligible individuals accessing care during the elimination program period, the mean (SD) age was 36.0 (13.5) years and 55.9% were women. From the pre–elimination program period to the elimination program period, first-time HCV screening coverage increased from 20.9% to 38.2%, and identification of current HCV infection and treatment in newly screened individuals increased from a mean (SD) of 170 (40) per year to 244 (4) per year and a mean of 95 (133) per year to 215 (9) per year, respectively. During the implementation period, of the 793 individuals with current HCV infection accessing the CNHS, 664 were evaluated (83.7%), 394 (59.3%) initiated treatment, and 335 (85.0%) had documented cure. In less than 2 years, the 85% 3-year goal was reached for cure (85.0%), and the goal for linkage to care was nearly reached (83.7%), whereas screening (44.1%) and treatment initiation (59.3%) required more time and resources.

**Conclusions and Relevance:**

This cohort study found that after 22 months of implementation, the CNHS community-based HCV elimination program was associated with an improved cascade of care. The facilitators and lessons learned in this program may be useful to other organizations planning similar programs.

## Key Points

**Question:**

Can a community-based tribal hepatitis C virus (HCV) elimination program be implemented successfully in a rural setting?

**Findings:**

In this cohort study, first-time HCV screening coverage increased from 20.9% to 38.2% from 3 years before to 22 months into implementation of the Cherokee Nation Health Services HCV elimination program. Identification, treatment, and cure of newly identified current HCV infections increased.

**Meaning:**

In this study, implementation of a community-based HCV elimination program was associated with an improved cascade of care; this information may serve other organizations planning to implement similar programs in large rural areas.

## Teaser

This cohort study evaluates the first 22 months of implementation of the Cherokee Nation Heath Services community-based program designed to eliminate hepatitis C virus (HCV) infection.

## Introduction

In 2019, hepatitis C virus (HCV) infection was the leading cause of death among 60 notifiable infectious diseases in the US and contributed to increasing rates of liver-related mortality.^1,2,3^ This infection disproportionately affects American Indian and Alaska Native individuals, with an estimated incidence of 2.9 per 100 000 population, more than twice that of all other racial/ethnic groups^1,4^; the rate of HCV-related deaths in this population is 10.2-fold higher than that in the general US population.^5^

The Cherokee Nation Health Services (CNHS), the largest tribal health system in the US, provides care to American Indian and Alaska Native individuals residing in rural northeast Oklahoma. After the CNHS began screening individuals born between 1945 and 1965, 388 patients tested positive for HCV RNA through July 2015.^6,7^ This, combined with the reported HCV-related disparities for American Indian and Alaska Native individuals at the national level, prompted the CNHS to develop the first, to our knowledge, community-based HCV elimination program in the US.^1,4^

The primary objective of this study was to evaluate the association of the CNHS HCV elimination program with each element of the cascade of care (screening, linkage to care, treatment, and cure) through the first 22 months of implementation. Secondary objectives were to compare screening, treatment, cure, and detection of HCV infection by birth cohort and sex before (October 1, 2012, to October 31, 2015) and 22 months after (November 1, 2015, to August 31, 2017) initial implementation of the program and to describe barriers and enablers of the elimination program.

## Methods

This cohort study used data from the CNHS. The CN is a federally recognized, sovereign tribal nation with more than 380 000 tribal citizens worldwide and a reservation spanning all or part of 14 rural counties encompassing approximately 18 000 square kilometers in Oklahoma (eFigure 1 in the Supplement). The CNHS delivers health-related services to approximately 132 000 American Indian and Alaska Native individuals at the WW Hastings hospital in Tahlequah, Oklahoma, and 8 satellite clinics.^8^ Eligible individuals choose to have all, some, or none of their medical needs provided within the CNHS. The target and study populations were American Indian and Alaska Native individuals aged 20 to 69 years who accessed any CNHS service at least once from October 1, 2012, to August 31, 2017. Research components, including waiver of informed consent, were reviewed and approved by the CN and University of Oklahoma Health Science Center institutional review boards. This study used deidentified electronic health record (EHR) data, so it was not possible to obtain informed consent and a waiver of informed consent was obtained. This study followed the Strengthening the Reporting of Observational Studies in Epidemiology (STROBE) reporting guideline.

### HCV Elimination Program Activities

A comprehensive HCV elimination program was implemented with 3-year goals to screen 85% of the eligible individuals, perform HCV RNA testing for 85% of seropositive individuals, link 85% of those with current infection to care, treat 85% of those linked to care, and document cure for 85% of those treated. These 3-year targets were set before the 2016 national and international HCV elimination goals.^7,9,10^ A summary of the HCV elimination program activities implemented at both the community and the individual levels can be found below, with details in eTable 1 in the Supplement.

The CNHS infectious diseases team worked with CN leadership to secure the commitment of CNHS and CN leadership to the program. A multimedia public awareness campaign was developed in conjunction with the CN and the Oklahoma State Department of Health.

Several HCV screening strategies were implemented sequentially to universally screen individuals aged 20 to 69 years, including laboratory-triggered screening in the hospital and system-wide EHR screening reminders (eMethods in the Supplement). At the hospital, screening was conducted using third-generation enzyme immunoassay screening tests. In-house OraQuick HCV rapid tests (OraSure Technologies, Inc) were used on blood samples in satellite clinics and for point-of-care testing in dental clinics (eMethods in the Supplement). Providers received face-to-face education about the new screening policy, screening procedures, and using the EHR HCV screening reminders.

Reflex HCV polymerase chain reaction testing was performed on seropositive blood samples at the hospital, but this testing was not available at the satellite clinics. Individuals with current infection were linked to care and treated according to guidelines current at the time.^11^ A CNHS public health nurse conducted a home visit if individuals were unreachable by telephone or mail; if they were not present, they were considered lost to follow-up.

Capacity for evaluating and treating individuals with current infection was increased by expanding the Extension for Community Health Outcomes (ECHO) program^12^ and by means of on-site workshops, online HCV modules,^13^ preceptorships, and specialist support via telephone and email. Pharmacists throughout the CNHS were trained to treat HCV infection using a collaborative practice agreement and the ECHO program under the direction of the specialist. A case manager helped obtain direct-acting antivirals (DAAs) through insurance or patient-assistance programs. Individuals initiating treatment but not returning for 3 consecutive confirmed appointments were deemed lost to follow-up. Harm-reduction strategies included medication-assisted treatment for opioid use disorder and treatment as prevention for individuals with HCV infection who injected drugs within the previous 12 months.

### Pre–Elimination Program Period Activities

In October 2012, the CNHS adopted the Centers for Disease Control and Prevention HCV recommendations to screen all persons born from 1945 to 1965 in addition to high-risk populations.^7,9^ This recommendation was implemented in 2013 and was followed by implementation of an HCV ECHO program in July 2014.^12^ In addition, an HCV treatment database was developed in 2012. In late 2014, HCV training was expanded to the family residency program, and the HCV elimination program plan was presented to and approved by the CNHS governing board (eMethods and eTable 2 in the Supplement).

### Sources of Data and Measurements of Outcomes

The HCV antibody and RNA EHR data were provided by the CNHS from October 1, 2012, through August 31, 2017, for these analyses. The HCV treatment data from 2012 onward were obtained from the CNHS specialty clinic database. Anti-HCV seropositivity was defined as a positive antibody detection test result. Current HCV infection was defined as detection of HCV RNA or HCV genotype recorded in the EHR. The EHRs of all anti-HCV–positive, HCV RNA–positive, or HCV genotype–positive individuals were reviewed to verify current HCV infection, evaluation for treatment, treatment initiation, and cure. Linkage to care was defined as having a face-to-face HCV evaluation with a DAA-prescribing provider or HCV treatment pharmacist. Hepatitis C virus cure was defined as sustained virologic response 12 weeks after treatment discontinuation. Cure data were recorded through April 15, 2018.

### Statistical Analysis

The cumulative incidence of first-time screening among the population accessing care at the CNHS at least once and of newly detected anti-HCV seropositivity and current HCV infections (defined as HCV RNA positive) among those newly screened were estimated for each period (37 months in the pre–elimination program period and 22 months in the elimination program period). Cumulative incidence of newly identified anti-HCV seropositivity and current infection within each period were estimated by birth cohort and sex, with comparisons made using χ^2^ tests.

The cascade of care analysis included all HCV RNA–positive American Indian and Alaska Native individuals aged 20 to 69 years who accessed the CNHS between November 1, 2015, and August 31, 2017, regardless of screening history. Evaluation, treatment, and cure elements of the cascade of care included HCV RNA–positive individuals regardless of initial HCV diagnosis date, including those screened elsewhere and those ultimately receiving HCV treatment outside the CNHS. Individuals were excluded if they cleared HCV RNA spontaneously or through treatment initiated before November 1, 2015, or if they died before August 31, 2017, without receiving treatment. Cure proportions were estimated as intention to treat and per protocol treatment (eMethods in the Supplement).

Birth cohort estimates were limited to those born between 1945 and 1965 (older birth cohort) and 1966 and 1997 (younger birth cohort). The cumulative incidence of HCV infection in the CNHS population was estimated using bayesian analysis and conducted with WinBugs, version 1.4.3.^14^ Logistic regression was performed using SPSS, version 19 (IBM Corp) for the cascade of care estimates (eMethods in the Supplement).

## Results

A total of 74 039 American Indian and Alaska Native individuals aged 20 to 69 years accessed the CNHS at least once during the elimination program, and 86 978 in this same age group accessed care at least once during the pre–elimination program period. The mean (SD) age of these individuals was 36.0 (13.5) years during the elimination program period and 36.7 (13.8) years in the pre–elimination program period; 55.9% and 56.1% were women, respectively.

### Cascade of Care During the Elimination Program Period

**Figure 1.**
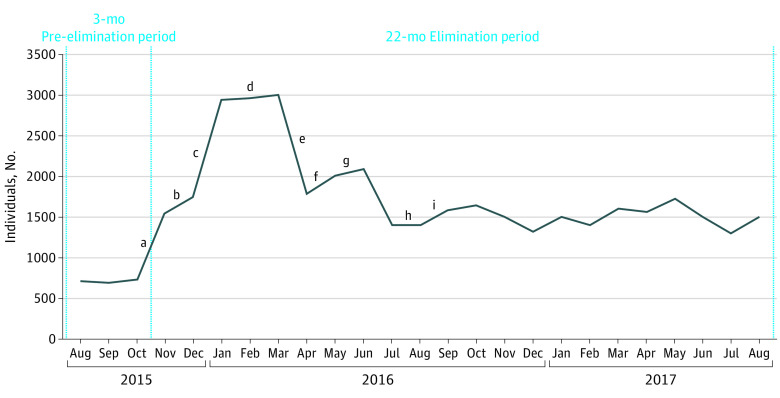
Individuals Aged 20 to 69 Years Screened for Hepatitis C Virus (HCV) Antibodies During the 3 Months Preceding the Elimination Program and During the Elimination Period ^a^The universal screening policy was implemented at the Cherokee Nation Health Services (CNHS) on November 1, 2015. ^b^Laboratory-triggered screening (LTS) was initiated at WW Hastings (WWH) Hospital in December 2015. ^c^Use of in-house tests for HCV antibody screening was initiated at satellite clinics on January 1, 2016. ^d^Point-of-care testing was initiated at WWH Hospital dental clinic in March 2016. ^e^Discontinuation of LTS occurred on March 31, 2016. ^f^Resumption of LTS with consent to treat on file occurred on May 19, 2016. ^g^Point-of-care testing was initiated at 1 satellite dental clinic in June 2016. ^h^Use of electronic health record reminders in the new CNHS electronic health record system was initiated on July 19, 2016 (LTS with consent to treat on file was discontinued). ^i^Physician training on use of the new EHR reminder occurred in July and August 2016. Point-of-care testing was implemented at WWH Hospital Behavioral Health Clinic in August 2016 but was not implemented systematically.

Among the 74 039 individuals accessing care, 32 684 (44.1%) were screened overall, with 20 683 (38.2%) screened for the first time among the unscreened population of 54 155. The largest increases in the number of screenings occurred after implementation of universal screening at the hospital (from 269 in October 2015 to 1228 in November 2015) and universal in-house screening in satellite clinics (from 1464 in December 2015 to 2845 in January 2016). Increases also occurred after implementation of laboratory-triggered screening (from 1228 in November 2015 to 1464 in December 2015) and after implementation and training on the screening reminder in the new EHR system (from 1054 in July 2016 to 1264 in September 2016); however, a large decrease occurred when the laboratory-triggered screening was discontinued on March 31, 2016 (from 2922 in March 2016 to 1493 in April 2016) (Figure 1 and eFigure 2 in the Supplement).

Among individuals screened for the first time during the elimination program period, 655 (3.2%) had positive test results, and 557 (85.0%) of those had an RNA or genotype test recorded; 447 (80.3%) were positive for HCV RNA. An additional 92 individuals with a positive result of an antibody test done outside the CNHS had newly detected HCV RNA at the CNHS, for a total of 539 current HCV infections.

### Linkage to Care, Treatment, and Cure

**Figure 2.**
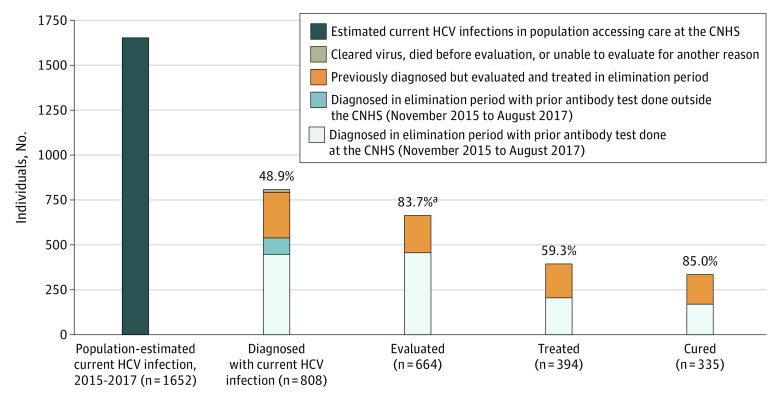
Cascade of Care Among Individuals Accessing the Cherokee Nation Health Services (CNHS) at Least Once With Estimated and Diagnosed Current Hepatitis C Virus (HCV) Infection During the Elimination Period The first bar represents the estimated number of people with current HCV infection in the CNHS population who accessed care at least once from November 1, 2015, to August 31, 2017 (n = 1652), not only those screened as reported in Table 1 and in the eMethods in the Supplement. The second bar represents individuals with current HCV infection (n = 808), including 447 individuals newly diagnosed during the elimination period, 92 individuals newly diagnosed in the elimination period with a previous antibody test outside the CNHS, 254 individuals diagnosed before November 2015, and 15 individuals who were identified but who could not be evaluated and followed-up because they either cleared the virus (n = 7), or died before evaluation (n = 2), were not identified (n = 1), or were not Native American or Alaska Native individuals (n = 5). The third bar represents individuals evaluated (n = 664) during the elimination period, including those diagnosed during the elimination period (n = 456) and those diagnosed with current infection before November 1, 2015 (n = 208). The proportion evaluated was calculated as the number of individuals evaluated (n = 664) divided by the number of individuals seen at the CNHS who were diagnosed with current HCV infection and accessed care at least once during the elimination period (n = 793) but does not include the 15 individuals who could not be followed up as specified above. The fourth bar represents individuals treated during the elimination program period (n = 394), including individuals diagnosed during the elimination program period (n = 205) and those diagnosed before November 2015 (n = 189). The fifth bar represents individuals cured under the intent to treat approach during the elimination program period (n = 335), including individuals diagnosed during the elimination program period (n = 170) and those diagnosed before November 2015 (n = 165).

**Figure 3.**
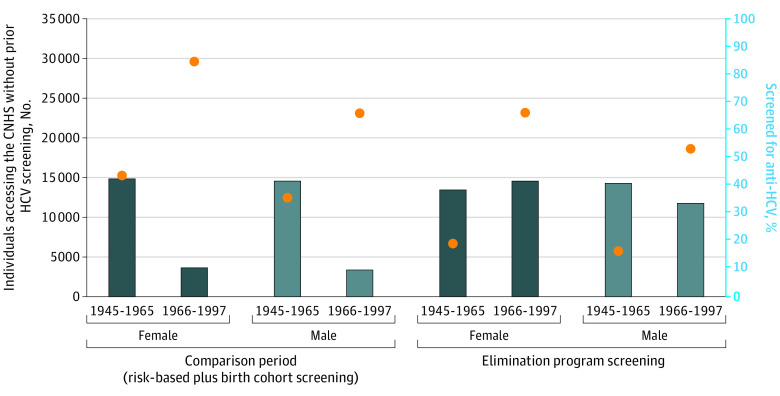
American Indian and Alaska Native Individuals Aged 20 to 69 Years Accessing Cherokee Nation Health Services (CNHS) Care Who Had No Prior Hepatitis C Virus (HCV) Screening and Newly Screened Individuals in Each Period by Sex and Birth Cohort The orange dots correspond to numbers of individuals accessing care at the CNHS who were not yet screened in each respective period.

During the elimination program period, an estimated 1652 individuals accessing the CNHS had current HCV infection (Figure 2), of whom 808 (48.9%) had a known diagnosis. Among the 793 who were still living and did not clear the infection, 539 (68.0%) received a new diagnosis during the elimination program period, whereas 254 (32.0%) had previously received a diagnosis but were not treated (Figure 3). Of the 793 currently infected individuals, 664 (83.7%) were evaluated; DAA treatment was initiated for 394 (59.3%) of the evaluated individuals, and 335 (85.0%) achieved a documented sustained virologic response 12 weeks after treatment discontinuation in the intention-to-treat analysis. In the per protocol treatment analysis, the cure rate was 96.5%, which did not vary by birth cohort, sex, genotype, or fibrosis severity (eTables 3-5 in the Supplement). Reasons for not achieving sustained virologic response 12 weeks after treatment discontinuation included loss to follow-up (n = 37), relapsed infection (n = 18), and death (n = 4).

### Comparison of Elimination and Pre–Elimination Program Periods

Figure 3 shows the number of individuals seen without previous screening and the cumulative incidence of first-time screening by birth cohort and period. First-time screening coverage remained high among the older birth cohort (38.2% among women and 40.5% among men) and more than tripled (10.4% to 41.4% among women and 9.8% to 33.5% among men) in the younger birth cohort during the elimination program period. The cumulative incidence of first-time screening increased from 20.9% in the pre–elimination program period to 38.2% in the elimination program period despite the shorter follow-up time in the latter period. In addition, the mean (SD) numbers of individuals identified with current HCV infection increased from 170 (40) per year in the pre–elimination program period to 244 (4) per year in the elimination program period; the mean (SD) number of individuals treated increased from 95 (133) per year to 215 (9) per year during these periods, respectively (Table 1).

**Table 1.** Comparison of HCV Screening, Newly Detected HCV Infections, Treatment, and Cure in the Pre–Elimination Program and Elimination Program Periods of the CNHS HCV Elimination Program

Variable	37-mo Pre–elimination program period^a^	22-mo Elimination program period^b^
HCV screening, No. (%)	37 915 (37.0)	32 684 (44.1)
First-time HCV screening, No. (%)	17 127 (20.9)	20 683 (38.2)
Newly detected HCV infections, total No. (mean annual No. [SD])	525 (170 [40])	447 (244 [4])
HCV-infected individuals treated, total No. (mean annual No. [SD])	301 (95 [133])	394 (215 [9])
Patients cured, No./total No. (%)		
ITT	254/301 (84)	335/394 (85)
PPT	254/270 (94)	335/353 (95)

Abbreviations: CNHS, Cherokee Nation Health Services; HCV, hepatitis C virus; ITT, intention to treat; PPT, per protocol treatment.

^a^ October 1, 2012, to October 31, 2015.

^b^ November 1, 2015, to August 31, 2017.

Using bayesian estimates, among those screened during the elimination program period, the 22-month cumulative incidence of newly detected anti-HCV seropositivity was higher among older adults (4.7%) than among younger adults (2.7%). Similarly, the estimated 22-month cumulative incidence of newly detected current HCV infection was higher among older adults (2.9%) than among younger adults (1.9%) (Table 2). In contrast, younger adults had higher 37-month cumulative incidences of newly detected anti-HCV seropositivity and current HCV infection during the pre–elimination program period compared with older adults.

**Table 2.** Cumulative Incidence of Newly Identified Anti-HCV Seropositivity and Current HCV Infections Among Newly Screened Individuals During the Pre–Elimination Program and Elimination Program Periods Overall and by Birth Cohort^a^

Screening period	Anti-HCV seropositivity			Current HCV infection		
	Birth cohort		Overall	Birth cohort		Overall
	1945-1965	1966-1997		1945-1965	1966-1997	
37-mo Pre–elimination program period^b^						
Individuals, No.	457	368	825	253	272	525
Estimated cumulative incidence (95% BCI)	3.9 (3.6-4.3)	6.9 (6.2-7.6)	4.8 (4.5-5.2)	2.2 (1.9-2.4)	5.1 (4.5-5.7)	3.1 (2.8-3.3)
22-mo Elimination program period^c^						
Individuals, No.	225	430	655	139	308	447
Estimated cumulative incidence (95% BCI)	4.7 (4.1-5.3)	2.7 (2.5-3.0)	3.2 (2.9-3.4)	2.9 (2.5-3.4)	1.9 (1.7-2.2)	2.2 (2.0-2.4)

Abbreviations: BCI, bayesian credible interval; CNHS, Cherokee Nation Health Services; HCV, hepatitis C virus.

^a^ Bayesian estimates included posterior median number of HCV frequencies and cumulative incidence with 95% bayesian credible intervals among the population accessing care at least once at CNHS during the period.

^b^ Risk-based plus older birth cohort screening (37 months).

^c^ Screening all individuals aged 20 to 69 years (22 months).

### Barriers and Enablers

An enabler of this program was the formal recognition by the CN leadership in July 2015 of HCV as a public health threat and a commitment to eliminate it from the CNHS, including a public proclamation by the Principal Chief designating October 30 as Hepatitis C Awareness Day. In November 2015, the CNHS expanded HCV screening to all individuals aged 20 to 69 years and expanded treatment through the ECHO program expansion. Political and CNHS leaders supported and attended a kick-off event to educate health care providers about HCV infection and the elimination program. Another enabler was the multimedia public awareness campaign implementation, starting in September 2016 (eResults in the Supplement). In addition, leadership was supportive of implementing harm reduction, resulting in delivery of medication-assisted treatment to 145 individuals with opioid use disorder and distribution of naloxone to prevent deaths caused by opioid overdose.

Although DAAs were provided through patient assistance programs, Medicaid, or private insurance, delays in DAA access owing to a long and complicated process to obtain them represented a barrier to treatment initiation. Moreover, when this program was implemented, third-party payers restricted DAA access for individuals with substance use disorder. Of the 394 individuals who initiated treatment, 52 had injected drugs within the previous 12 months. The proportions of intention-to-treat and per protocol treatment cure in this population were 87% and 96%, respectively, suggesting that substance use disorder was not a barrier to cure. To help further reduce the infection risk in this population, CNHS policy changes were discussed, and CN policy makers were supportive. However, Oklahoma laws create barriers to implementing syringe service programs. Another important barrier was the discontinuation of laboratory-triggered screening because of concerns that it was not being offered as an opt-out test despite requiring patients to have a general written consent document on file to qualify; this resulted in a reduction in the number screened.

## Discussion

This cohort study analyzed the first, to our knowledge, community-based HCV elimination program in the US that was implemented by the CNHS. Over the first 22 months of the program, 44.1% of eligible individuals who accessed care at the CNHS were screened, and most of the individuals diagnosed with current HCV infection were linked to care. The 3-year 85% program goals were achieved or nearly achieved for HCV cure and linkage to care, respectively, and considerable improvements from the previous period were made in HCV screening. Of note, unlike other elimination programs,^15,16,17,18^ direct funding for this program did not cover DAA costs.

Expanding HCV screening in a large, rural health care system is challenging. Nevertheless, improvements in HCV screening coverage, identification of individuals with current HCV infection, linkage to care, and treatment completion showed the potential effectiveness and feasibility of this elimination program and universal screening, which were implemented before recent Centers for Disease Control and Prevention and US Preventive Services Task Force recommendations for universal screening were published.^19,20^ The proportion of individuals screened in the CNHS elimination program was not as high as that in other programs.^21^ Nevertheless, the CNHS elimination program targeted all individuals in the CNHS population, including individuals who accessed the CNHS only once, even if the service accessed did not offer HCV screening (eg, physical therapy, drug refills, or optometry), and can therefore be viewed as a community-based program. The combination of laboratory-triggered screening at the hospital and screening at satellite clinics was associated with the largest increase in the number of individuals newly screened. However, to achieve all programmatic goals, it will be necessary to expand screening to CNHS sites where it is not offered and to optimize screening and downstream linkage to care where screening is occurring.

Although methods differ among studies, the 22-month cumulative incidence of anti-HCV seropositivity of 3.2% identified during the HCV elimination program period is higher than the US population estimates of 1.5%^22^ and is within the range of most studies conducted in American Indian and Alaska Native communities in the US (1.49%-7.04%).^23,24,25,26^ The 22-month cumulative incidence of newly detected anti-HCV seropositivity and current HCV infection in the CNHS were similar to Oklahoma prevalence estimates (3.2% vs 3.3% and 2.2% vs 1.8%, respectively), which are among the highest nationally.^22^ However, because the CNHS elimination program included enhanced screening, which likely detected more cases, and covered a period of 22 months, these estimates cannot be compared directly.

Improvements in linkage to care were associated with multiple efforts, including expanding the HCV-competent primary care work force. However, the relative contributions of each intervention were not studied. Analysis of the cascade of care revealed a substantial gap from HCV evaluation to treatment initiation. Contributing factors included no immediate access to DAAs and the time and processes required to obtain them, individuals prioritizing other medical problems over HCV infection, and socioeconomic factors. Addressing root causes of this gap and optimizing treatment initiation are critical.

The per protocol treatment and intention-to-treat cure rates were similar to those found in clinical trials^27,28^ and reports from other HCV elimination programs,^16^ highlighting the effectiveness of primary care providers using the Project ECHO model to evaluate HCV and DAAs for treatment of individuals with current HCV infection.^29^ The lower intention-to-treat cure rate estimate may reflect individuals not returning for testing to assess whether they achieved sustained virologic response 12 weeks after treatment discontinuation. This finding suggests that improvement of care retention at this point or not requiring sustained virologic response 12 weeks after treatment discontinuation after intention-to-treat and per protocol care rates become comparable is needed. As previously reported,^30,31^ these cure rates among individuals actively injecting drugs were similar to those among individuals not actively injecting, supporting the practice of HCV treatment in this population.

Encouraging outcomes of large-scale HCV elimination and control programs from Iceland, the Republic of Georgia, Egypt, Australia, and the US Veterans Affairs National Health Care System have been reported.^15,16,17,18,32^ The Icelandic HCV elimination program reported high rates of treatment initiation (93.5%), completion (91.3%), and cure (96%) based on treatment as prevention and prioritizing treatment for people who inject drugs, individuals with advanced liver diseases, and prisoners.^17^ This and other programs^16^ provided immediate access to DAAs, whereas the CNHS HCV elimination program did not, resulting in long approval waiting periods, likely contributing to lower treatment initiation rates. Nevertheless, these programs have some factors in common with the CNHS program, including political commitment, public awareness campaigns, screening expansion, primary care involvement in HCV care, ultimate access to DAAs, and minimization of barriers to treatment for those with infection. Harm-reduction services also vary across programs, from robust services in Iceland and Australia to incipient services in the CNHS. However, the changes seen in the CNHS from the pre–elimination program period to the elimination program period suggest that this program was associated with improvements in the number of individuals screened, diagnosed, evaluated, and cured.

### Limitations

This study has limitations. Estimating the population eligible for screening is challenging; however, this study estimated the cumulative incidence of HCV infection based on screening results for individuals accessing a health system rather than being a population-based study. We used EHRs to evaluate who had accessed care at least once but had not been previously screened as the denominator to estimate first-time screening coverage. However, some individuals accessed the CNHS services at centers where screening was not offered, and others accessed the CNHS infrequently with little opportunity to be screened. Nonetheless, our screening coverage estimates likely reflect the percentage of the American Indian and Alaska Native population accessing care at the CNHS. In addition, EHR data have some limitations, including difficulties tracing individuals through the system. To mitigate this factor, data were gathered from 2 EHR systems and from the reference laboratory. All individuals with a current infection also had their EHRs checked manually to verify the data’s accuracy.

## Conclusions

In this cohort study, implementation of the CNHS community-based HCV elimination program was associated with an improved cascade of care. The program achieved 2 of the 3-year targets in less than 2 years and identified enablers and barriers that could serve as a model for other elimination programs. Although screening coverage improved substantially among those accessing the CNHS, next steps might include community outreach programs targeting at-risk populations and individuals not accessing or minimally accessing the health care system. Moreover, harm-reduction services were successful and should be prioritized to interrupt HCV transmission. In addition, identification of systems to increase DAA availability may be associated with improved linkage to care and treatment rates.

## Notes

Supplement. **eMethods.** **eFigure 1.** Map of Cherokee Nation reservation and Cherokee Nation Health Services Facilities **eTable 1.** Cherokee Nation Health Services (CNHS) activities during the Hepatitis C Virus (HCV) elimination period, November 2015 – August 2017 **eTable 2.** Cherokee Nation Health Services (CNHS) Hepatitis C Virus (HCV) activities in the Pre-elimination program period, October 1, 2012-October 31, 2015 **eResults.** **eFigure 2.** Number of individuals accessing care at CNHS without previous HCV screening and percentage of those screened for HCV with universal screening during the elimination program period by screening strategy, location, and birth cohort **eTable 3.** Individual demographics and clinical characteristics of patients initiating treatment during the elimination program period, November 1, 2015-October 31, 2017, overall and by birth cohort **eTable 4.** Cure proportions during the elimination program period (November 2015-August 2017) for individuals who completed per protocol treatment stratified by birth cohort and gender, genotype, and liver fibrosis **eTable 5.** Crude odds ratios (OR) of HCV cure by gender, genotype, and fibrosis severity among individuals who completed per protocol treatment CNHS from November 2015 through August 2017
