# Supplementary material for: Evaluation of the Cherokee Nation Hepatitis C Virus Elimination Program in the First 22 Months of Implementation
Source: JAMA Netw Open. 2020 Dec 18;3(12):e2030427. doi: 10.1001/jamanetworkopen.2020.30427 (PMC7749444; doi:10.1001/jamanetworkopen.2020.30427)
Supplement: Supplement. — eMethods. eFigure 1. Map of Cherokee Nation reservation and Cherokee Nation Health Services Facilities eTable 1. Cherokee Nation Health Services (CNHS) activities during the Hepatitis C Virus (HCV) elimination period, November 2015 – August 2017 eTable 2. Cherokee Nation Health Services (CNHS) Hepatitis C Virus (HCV) activities in the Pre-elimination program period, October 1, 2012-October 31, 2015 eResults. eFigure 2. Number of individuals accessing care at CNHS without previous HCV screening and percentage of those screened for HCV with universal screening during the elimination program period by screening strategy, location, and birth cohort eTable 3. Individual demographics and clinical characteristics of patients initiating treatment during the elimination program period, November 1, 2015-October 31, 2017, overall and by birth cohort eTable 4. Cure proportions during the elimination program period (November 2015-August 2017) for individuals who completed per protocol treatment stratified by birth cohort and gender, genotype, and liver fibrosis eTable 5. Crude odds ratios (OR) of HCV cure by gender, genotype, and fibrosis severity among individuals who completed per protocol treatment CNHS from November 2015 through August 2017 [file jamanetwopen-e2030427-s001.pdf]

Supplemental Online Content

Mera J, Williams MB, Essex W, et al. Evaluation of the Cherokee Nation hepatitis C virus elimination program in the first 22 months of implementation. *JAMA Netw Open*. 2020;3(12):e2030427. doi:10.1001/jamanetworkopen.2020.30427

eMethods.

eFigure 1. Map of Cherokee Nation reservation and Cherokee Nation Health Services Facilities

eTable 1. Cherokee Nation Health Services (CNHS) activities during the Hepatitis C Virus (HCV) elimination program period, November 2015 – August 2017

eTable 2. Cherokee Nation Health Services (CNHS) Hepatitis C Virus (HCV) activities in the pre-elimination program period, October 1, 2012-October 31, 2015

eResults.

eFigure 2. Number of individuals accessing care at CNHS without previous HCV screening and percentage of those screened for HCV with universal screening during the elimination program period by screening strategy, location, and birth cohort

eTable 3. Individual demographics and clinical characteristics of patients initiating treatment during the elimination program period, November 1, 2015-October 31, 2017, overall and by birth cohort

eTable 4. Cure proportions during the elimination program period (November 2015-August 2017) for individuals who completed per protocol treatment stratified by birth cohort and gender, genotype, and liver fibrosis

eTable 5. Crude odds ratios (OR) of HCV cure by gender, genotype, and fibrosis severity among individuals who completed per protocol treatment CNHS from November 2015 through August 2017

This supplemental material has been provided by the authors to give readers additional information about their work.

© 2020 Mera JR et al. *JAMA Network Open.*

eMethods

Cherokee Nation Reservation and the geographic distribution of the Cherokee Nation Health Services (CNHS) facilities, including the WWH hospital and eight Ambulatory Health Care Centers, satellite clinics in the manuscript, are displayed in eFigure 1.[1]

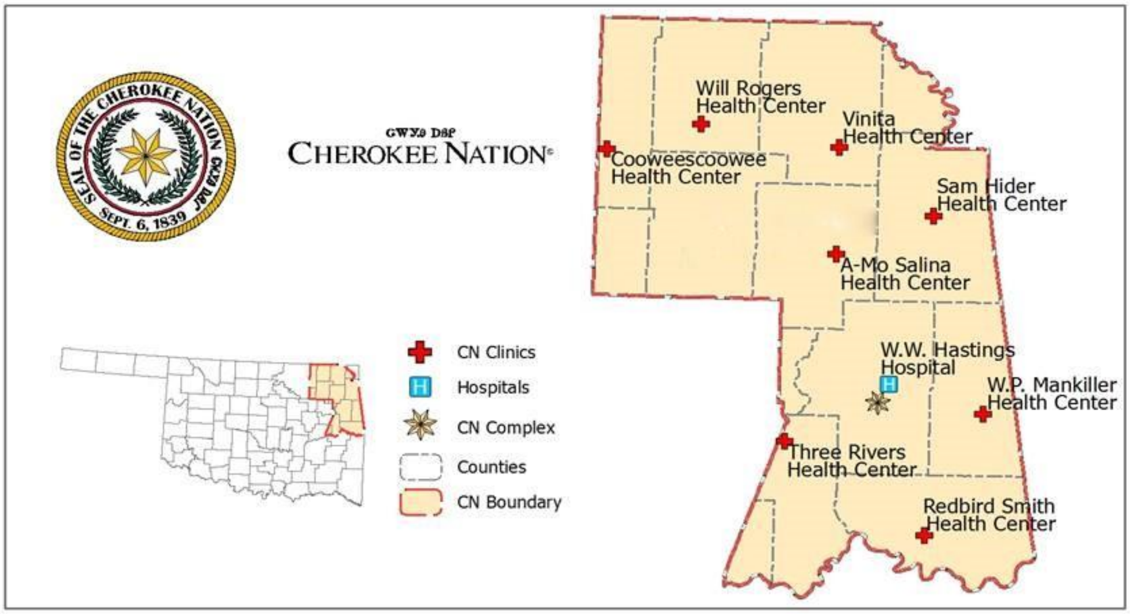

eFigure 1. Map of Cherokee Nation (CN) Health Services facilities, including the WWH hospital and eight satellite clinics, in the 14-county reservation of Cherokee Nation. (Map courtesy of Cherokee Nation)

Details of CNHS HCV screening strategies and treatment activities implemented by period

Universal screening was initiated on November 1, 2015 as part of the CNHS elimination program. Universal screening included a one-time screen for all individuals aged 20-69 years who accessed care at CNHS. Various screening strategies were implemented at WWH hospital and in the satellite clinics to achieve universal screening during the elimination program period, November 1, 2015-August 31, 2017. These strategies, presented the main manuscript, are described in more detail below and in eTable 1.

WWH hospital implemented a laboratory-triggered screening (LTS) process in December of 2015. The LTS process involved collecting an additional tube of blood for HCV antibody testing from all individuals aged 20-69 years who were undergoing phlebotomy for any other reason. On March 31, 2016 LTS was discontinued because there were concerns that it did not allow for an “opt out process”. LTS was resumed on May 19, 2016 with the condition that a patient had an up-to-date consent for treatment form on file in the CNHS EHR system. This form is a general medical care consent document that all individuals who access the CNHS have to sign to receive medical care and is renewed annually. This form states that the individual will be screened for HCV and HIV at the medical providers’ discretion with an option to opt out of these tests. In July 2016, an EHR HCV screening reminder in the new EHR system replaced the LTS with consent to treat on file approach. Under the default EHR settings, this HCV screening reminder was not automatically visible to providers during routine EHR system use; therefore, training was offered throughout CNHS in July and August 2016 to show providers where to find the reminders in the new EHR system. Point-of-care HCV rapid-test screening was implemented using OraQuick rapid HCV tests kits (OraSure Technologies, Inc) in March 2016 at the hospital dental clinic,

© 2020 Mera JR et al. *JAMA Network Open.*

while point of care testing was initiated at the WWH hospital Behavioral Health clinic in August 2016 but discontinued after one month due to logistical issues.

On-site HCV screening with OraQuick rapid HCV tests kits replaced send-out tests for universal HCV screening at all satellite clinics in January 2016. Rapid test kits were used in the clinic laboratory on blood samples collected through phlebotomy. Hence, the test results were not available during individuals' medical visit. If a test was positive, the individual was contacted and scheduled for RNA testing. Point-of-care HCV rapid screening using a finger prick was expanded to a second dental clinic located in a satellite clinic in June 2016.

eTable 1. Cherokee Nation Health Services (CNHS) activities during the Hepatitis C Virus (HCV) elimination program period, November 1, 2015 – August 31, 2017

Strategy	Activity	Outcome
**Develop program goals**	Assessment of goals feasibility	85% screening, link to care, and treatment goals established
**Secure Political Commitment**	Cherokee Nation (CN) HCV Awareness Day proclamation signed by Chief	Elimination program Implementation
**HCV Screening**	• Universal screening policy implemented<br>• Screening sites expanded and medical providers and ancillary staff trained[1]<br>• Multiple screening strategies implemented at WWH hospital, including laboratory-triggered screening, laboratory-triggered with consent-to-treat on file, and EHR reminders.	• 32,684 unique individuals aged 20-69 screened<br>• 20,683 unique individuals aged 20-69 screened for the first time<br>• 539 unique individuals with current HCV infection diagnosed in EHR<br>• HCV screening reminder in new EHR system implemented in July 2016
**Expand HCV clinical capacity for evaluation and treatment**	Recruit and train volunteer Primary Care Providers and Pharmacists using Project Extension for Community Healthcare Outcomes (ECHO)	• 11 PCPs recruited<br>• 6 pharmacists recruited<br>• 394 individuals treated
**Provider Education**	Training of providers in HCV evaluation and management	Ongoing training at recurring ECHO meetings
**Defining Disease Burden**	Establish anti-HCV seroprevalence estimate	655 unique individuals identified with anti-HCV seroprevalence
**Raising Awareness**	Public awareness campaign developed by third-party media company with OSDH[2]	Implemented in September of 2016
**Harm Reduction**	• Medically Assisted Treatment clinic started<br>• Syringe service programs	• 3 providers trained on MAT<br>• 145 individuals on MAT<br>• Meetings with CN leadership; not implemented due to Oklahoma laws preventing syringe service programs
**Research Activities**	Risk factor study<br>Cross sectional screening study	Completed<br>Completed

[1,]Screening sites were expanded at WWH hospital to include: the Obstetrics and Gynecology Department, Dental clinic, Urgent Care, Emergency Department, individuals admitted to the Medical Surgical floor and the intensive care unit. Individuals in the Urgent Care and Emergency department were screened systematically only during the period in which lab-triggered screening was implemented. The medical providers caring for patients received face-to-face training on the new HCV screening policy.

[2]OSDH: Oklahoma State Department of Health

© 2020 Mera JR et al. *JAMA Network Open.*

Pre-elimination Program Period: Risk-based plus birth-cohort period. Some HCV activities occurred at WWH hospital and in the satellite clinics during the pre-elimination program period, October 1, 2012-October 31, 2015. These activities are described in more detail below and in eTable 2.

An EHR HCV screening reminder was installed in July of 2013 and discontinued in July of 2015 when the EHR system was replaced. Installation of a HCV screening reminder in the new EHR was requested but it did not occur until July 19, 2016 (see above). The HCV ECHO program used telehealth technology[2] and online modules[3] to train and support CNHS primary care providers and pharmacists to develop knowledge in HCV management and treatment and enable them to deliver best practice HCV care. The CNHS HCV ECHO program initially consisted of seven HCV care providers. The program had a hub, physically located at WWH hospital, and four spokes located in the outlying clinics. The hub was composed of a lead clinician (Infectious Disease Specialist), a behavioral health psychologist, a case manager, an ECHO coordinator, and onsite Information Technology support. The spokes were staffed by a total of six clinicians, including two physicians, two nurse practitioners, and two clinical pharmacists, working at the four outlying clinics.

As part of the CNHS HCV ECHO, the spokes' providers participated in weekly HCV clinics via videoconference, called "Knowledge Networks", where they presented their cases by sharing individual medical histories, laboratory results, treatment plans, and questions about best practices and individual challenges. The hub team provided advice and clinical mentoring during these sessions. Additionally, these case-based discussions were supplemented with short didactic presentations to improve content knowledge.

eTable 2. Cherokee Nation Health Services (CNHS) Hepatitis C Virus (HCV) activities in the Pre-elimination Program Period, October 1, 2012-October 31, 2015.

Strategy	Activity	Outcome
Baby Boomer Birth Cohort Screening plus Risk-based screening*	Implemented Provider education<br>Electronic Health Record (EHR) reminder created	18,270 unique individuals aged 20-69 screened<br>566 unique individuals with current HCV infection diagnosed in EHR<br>CNHS providers and ancillary staff educated regarding HCV recommendations* and the use of EHR reminder
Train Primary Care Providers in HCV Treatment	Established HCV Extension for Community Healthcare Outcomes (ECHO) in CNHS	7 medical providers trained in HCV management using the Project ECHO model<br>301 individuals treated
Partnership Development	University of Oklahoma Health Sciences Center, Centers for Disease Control and Prevention, Oklahoma State Department of Health<br>Extramural funding pursued	Planning and development of a HCV Elimination Program grant: Grant awarded in 2015

* Centers for Disease Control and Prevention recommended HCV testing for those who: 1. Are currently injecting drugs or have ever injected drugs, including those who injected once or a few times many years ago; 2. Have certain medical conditions, including persons: who received clotting factor concentrates produced before 1987, were ever on long-term hemodialysis, with persistently abnormal alanine aminotransferase levels (ALT), or who have HIV infection; 3. Were recipients of transfusions or organ transplants before July 1992, including persons who: were notified that they received blood from a donor who later tested positive for HCV infection, or received a transfusion of blood, blood components, or an organ transplant; 4. Had a recognized exposure such as: healthcare, emergency medical, and public safety workers after needle sticks, sharps, or mucosal exposures to HCV-positive blood; and 5. Children born to HCV-positive women.

© 2020 Mera JR et al. *JAMA Network Open.*

Statistical analyses

For the CNHS HCV Cascade of Care, the number of people with current HCV infection was estimate for the whole CNHS population who had accessed care at least once between October, 1 2012-August 31, 2017 by multiplying the gender-specific cumulative incidence of current HCV infection calculated using the EHR data by the number of males and females accessing the CNHS from October 1, 2012 to August 31, 2017. The data gathered from the EHR on individuals with newly detected HCV RNA was used to calculate the percentages for each cascade of care element for each period. For these estimates, the date of first identification of current infection (i.e., first RNA test), the first date of evaluation, the date treatment was initiated, and the date SVR12 was reached were used to estimate the number of individuals in each cascade of care element. There were some individuals diagnosed in a previous period, but evaluated in a later period. Individuals evaluated during the elimination program period, regardless of their diagnosis date, were included in the evaluation, treatment, and cure elements of the cascade of care. Using the Infectious Disease clinic database, the proportion of individuals cured in the elimination program period was estimated according to the intent-to-treat (ITT) and a Per Protocol Treatment (PPT) approach analyses. The ITT cure proportion was estimated by dividing the number of individuals initiating treatment who attained SVR12 by all those who initiating treatment, irrespective of SVR12 data availability. The PPT proportion was estimated by dividing the number of individuals who attained SVR12 by the number of those who initiated treatment with SVR12 data available. Only individuals who had accessed the CNHS between October 1, 2012 and October 31, 2017 and had a documented HCV antibody or RNA in the EHR were included in these elements of the cascade of care. Cure proportions were evaluated up to April 15, 2018.

First-time screening coverage was calculated by dividing the number of newly HCV-screened individuals by the number of individuals eligible for HCV screening, defined as those aged 20-69 and accessing care at least once in CNHS during each period and not previously screened for HCV antibodies. In addition, the total proportion screened was calculated by dividing the total number of individuals screened during each period by all individuals accessing CNHS during that period, regardless of previous screening.

Bayesian analyses used a Beta distribution to estimate the number and cumulative incidence of anti-HCV seropositive individuals among those newly screened within the period. The cumulative incidence of newly detected anti-HCV cases was calculated by dividing the number of newly identified anti-HCV seropositive individuals among the total number of newly screened individuals during the period. The number of individuals with newly detected current HCV infection among seropositive individuals with missing HCV RNA test results was estimated by multiplying the proportion of newly detected current HCV infection among anti-HCV seropositive individuals tested for HCV RNA by the number of anti-HCV seropositive individuals without an HCV RNA test result. This estimate was added to the number of newly detected HCV RNA-positive individuals to obtain an estimated total number of individuals with current HCV infection among all newly screened. The cumulative incidence of current HCV infections for each period was assumed to follow a beta distribution with alpha parameter equal to the estimated total number of current HCV individuals +1 and beta parameter equal to the total number of individuals screened overall minus the estimated total number of current HCV individuals +1. The CNHS population estimates for the frequency and cumulative incidence were estimated by period and birth cohort. Additionally, since the two periods differed by number of months, the annual mean number of newly detected HCV infections were estimated within each period by summing the number of newly detected HCV infections for each month in that period, then dividing the resulting total by the number of months within that period and multiplying the result by 12.

Chi-square tests were used to compare estimates by birth-cohort and period. Chi-square tests estimates and 95% confidence intervals were generated in SAS 9.4. Cumulative incidence of anti-HCV and current HCV infection and corresponding 95% Bayesian Credible Intervals (95% BCI) were estimated using

© 2020 Mera JR et al. *JAMA Network Open.*

WinBUGS 1.4.3.[4] Vague priors were used for all proportions (i.e., beta distribution with parameters 1 and 1).

Logistic regression analyses were used to estimate the odds of being cured from HCV by demographic group, gender and age, HCV genotype, and severity of liver fibrosis, during the elimination program period. Logistic regression analyses were conducted using SPSS statistical software, version 19.

eResults.

Awareness Campaign

The multi-media public awareness campaign included out-of-home advertising, such as billboards and gas station pump toppers, and ran for eight weeks throughout the CN reservation. Indoor posters were continuously displayed in CNHS hospital and clinics. Radio advertising included 1,261 spots on local stations that ran for 21 days. CN website digital advertisements and social media marketing via the CN Public Health Facebook page ran for 8 days. The multi-media campaign had an estimated impact that included 12,255,000 impressions by out-of-home modalities, 1,300,000 impressions by radio spots, and 8,282,459 impressions by digital display. Social media marketing reached 64,815 individuals within the 10-targeted zip codes, with an average of 5.99 exposures per person and a 0.30% click-through rate. Among the 1,170 individuals making website clicks, 576 were 50-65 years old and 594 were 20-49 years old.

Expansion of the CNHS HCV ECHO Program

During the elimination program period the ECHO spokes increased from four to seven sites, covering all except one of the satellite clinics. The hub team expanded to include a clinical pharmacist, a nurse practitioner, and a dedicated DAA procurement case manager. During this process, 11 primary care providers and 6 pharmacists were recruited and trained in HCV care.

Screening coverage by site, screening strategy and birth cohort

The number of individuals accessing care at CNHS without prior screening at CNHS during the elimination program period and the percentage newly screened among the older and younger birth cohorts are displayed in eFigure 2 for those seen at the hospital (eFigures 2A and 2B) and at the satellite clinics (eFigures 2C and 2D). Among older and younger birth cohorts seen at the hospital, more individuals were screened during the LTS period (28.9% and 27.3%, respectively), followed by LTS with consent to treat on file (17.5% and 17.2%, respectively), than during the time period when LTS was discontinued (10.4% and 10.0%, respectively) or when the EHR reminder was re-established (11.2% and 11.9%, respectively). Among both birth cohorts seen at satellite clinics, the proportions screened were almost ten times higher after the satellite clinics obtained rapid test kits for the universal screening in January 2016.

© 2020 Mera JR et al. *JAMA Network Open.*

eFigure 2a: Hospital screening strategies among older adults (YOB: 1945-1965)

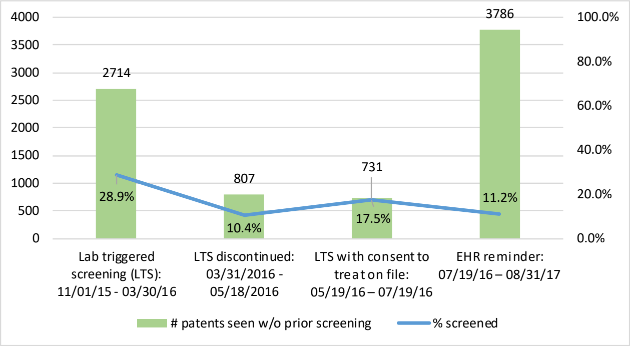

eFigure 2b: Hospital screening strategies among younger adults (YOB: 1966-1997)

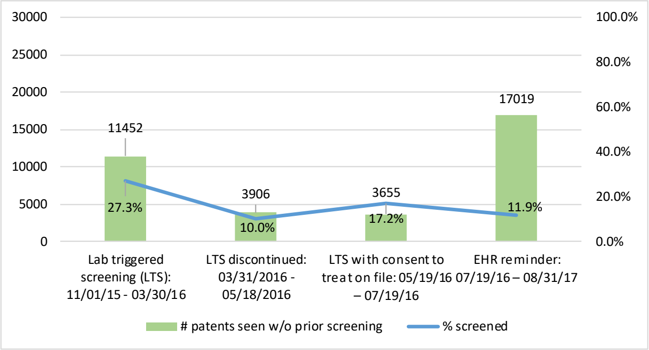

eFigure 2c: Satellite clinic screening among older adults (YOB: 1945-1965)

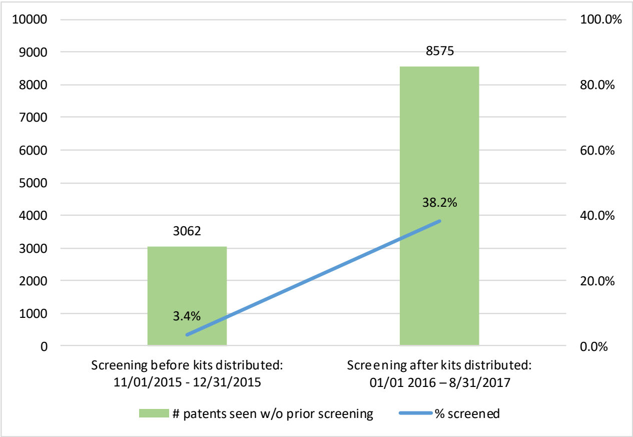

eFigure 2d: Satellite clinic screening among younger adults (YOB: 1966-1997)

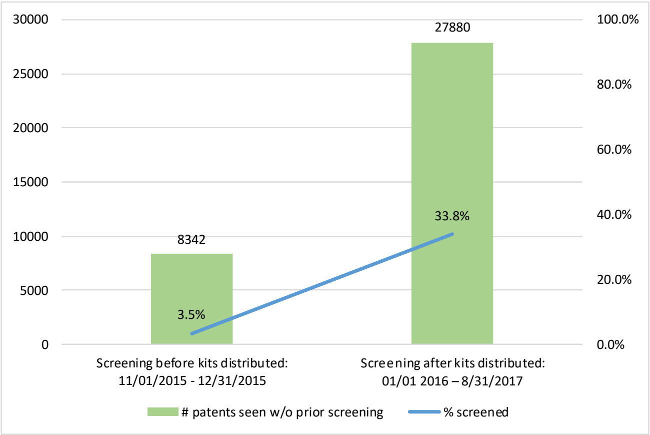

eFigure 2. Individuals accessing care at CNHS without previous HCV screening and the percentage of those screened for HCV with universal screening* during the elimination program period by screening strategy, location, and birth cohort at WWH hospital (eFigures 2A and 2B) and CNHS satellite clinics (eFigures 2C and 2D).

Cure Proportions Stratified by Birth Cohort, demographics and Liver Fibrosis Severity

Of those initiating treatment, 61% were male, with a median age of 47. The percentage of women initiating treatment was slightly higher among the younger birth cohort than the older birth cohort (41% vs 36%, eTable3). The majority of individuals had a HCV RNA genotype 1 (61%), followed by genotype 2 (22%), and genotype 3 (17%). Moderate and advanced liver fibrosis were detected in 29% and 11% of individuals, respectively. Among individuals with SVR12 available, PPT cure proportions were 97% (331/343) (eTables 3-4). In addition, no association was found between HCV cure and gender, genotype, or fibrosis severity (eTable 5).

© 2020 Mera JR et al. *JAMA Network Open.*

eTable 3. Individual demographics and clinical characteristics of patients initiating treatment during the elimination program period, November 1, 2015-October 31, 2017, overall and by birth cohort.

	Birth Cohort Evaluated, 11/1/2015 – 8/31/2017		
	**Total (%)**	**Born 1945-1965**	**Born 1966-1997**
**Demographics**			
**Individuals Initiating Treatment, N**	390[a]	194	196
**Age at treatment initiation, range, years**	20-69	50-69	20-51
**Median age at treatment initiation, years**	47	57	37
**Female sex, N (%)**	150 (39%)	69 (36%)	81 (41%)
**Male sex, N (%)**	240 (61%)	125(64%)	115 (59%)
**Genotype**			
**Genotype I, N (%)**	238 (61%)	113(58%)	125 (64%)
**Genotype II, N (%)**	87 (22%)	46 (24%)	41 (21%)
**Genotype III, N (%)**	65 (17%)	35 (18%)	30 (15%)
**Liver Fibrosis Severity**			
**Mild, N (%)**	233 (60%)	77 (40%)	156 (80%)
**Moderate, N (%)**	113 (29%)	82 (42%)	31 (16%)
**Severe, N (%)**	44 (11%)	35 (18%)	9 (4%)

[a] Four individuals were excluded from this analysis because they either had mixed genotypes (3 individuals) or had genotype 4 (1 individual)
[b] Liver fibrosis was determined with FIB-4 Score: Mild < 1.45, Moderate > 1.45 and < 3.25, Severe: > 3.25

© 2020 Mera JR et al. *JAMA Network Open.*

eTable 4. Cure proportions during the elimination program period (November 2015-August 2017) for individuals who completed per protocol treatment stratified by birth cohort and gender, genotype, and liver fibrosis.

	Period Totals			Born 1945-1965			Born After 1965		
	Total treated	SVR 12	Cured (N), PPT (%)	Total treated	SVR 12	Cured (N), PPT (%)	Total treated	SVR 12	Cured (N), PPT (%)
**Number of individuals treated**	**390**[a]	**343**	**331 (96.5%)**	**194**	**181**	**175**	**196**	**162**	**156**
**Female, N (%)**	150	133	126 (94.7)	69	66	62 (93.9)	81	67	64 (95.5)
**Male, N (%)**	240	210	205 (97.6)	125	115	113 (98.3)	115	95	92 (96.8)
**Genotype I, N (%)**	238	209	202 (96.7)	113	106	104 (98.1)	125	103	98 (95.1)
**Genotype II, N (%)**	87	75	73 (97.3)	46	42	40 (95.2)	41	33	33 (100.0)
**Genotype III, N (%)**	65	59	56 (94.9)	35	33	31 (93.9)	30	26	25 (96.2)
**Liver Fibrosis Severity**[b]									
**Mild, N (%)**	233	197	191 (97.0)	77	73	71 (97.3)	156	124	120 (96.8)
**Moderate, N (%)**	113	107	103 (96.3)	82	77	74 (96.1)	31	30	29 (96.7)
**Severe, N (%)**	44	39	37 (94.9)	35	31	30 (96.8)	9	8	7 (87.5)

a Four individuals were excluded from this analysis because they either had mixed genotypes (3 individuals) or had genotype 4 (1 individual).
b Liver fibrosis was determined with FIB-4 Score: Mild < 1.45, Moderate > 1.45 and < 3.25, Severe: > 3.25.

eTable 5: Crude odds ratios (OR) of HCV cure by gender, genotype, and fibrosis severity among individuals who completed per protocol treatment CNHS during the elimination program period (November 2015-August 2017).[a]

	Cured from HCV		Total with SVR12	Crude OR (95% CI)	P-Value
**Treated Individuals (N= 390)**[b]	**N= 331**[b]	**%**	**N = 343**[c]		
**Gender**					
**Male**	205	97.6	210	2.30 (0.71 – 7.39)	0.16
**Female (Ref)**	126	94.7	133	--------	--------
**Genotype**					
**Genotype 1**	202	96.7	209	1.55 (0.39 – 6.17)	0.54
**Genotype 2**	73	97.3	75	1.92(0.31 – 11.94)	0.48
**Genotype 3 (Ref)**	56	94.9	59	--------	--------
**Liver Fibrosis Severity**					
**Mild (FIB-4 < 1.45)**	191	97.0	197	1.67 (0.32 – 8.57)	0.54
**Moderate (FIB-4 1.46 – 3.24)**	103	96.3	107	1.34 (0.24 – 7.63)	0.74
**Severe (FIB-4 > 3.25) (Ref)**	37	94.9	39	--------	--------

[a] Only individuals who initiated treatment between 11/1/2015 and 8/31/2017 were included in these analysis but cure rates were evaluated through April 15, 2018.
[b] Four individuals were excluded from this analysis because they either had mixed genotypes (3 individuals) or had genotype 4 (1 individual
[c] The 343 individuals are the ones who had SVR 12 data available.

© 2020 Mera JR et al. *JAMA Network Open.*

Supplemental References

1. Cherokee Nation Health Services. Accessed November 12, 2020. https://health.cherokee.org/
2. Arora S, Kalishman S, Thornton K, et al. Expanding access to hepatitis C virus treatment—Extension for Community Healthcare Outcomes (ECHO) Project: disruptive innovation in specialty care. *Hepatology*. 1 2010;52(3):1124-1133. Medline:20607688 2 doi:10.1002/hep.23802.
3. University of Washington. Hepatitis C online. Accessed November 12, 2020. https://www.hepatitisc.uw.edu/
4. Lunn, D.J., Thomas, A., Best, N., and Spiegelhalter, D. (2000) WinBUGS — a Bayesian modelling framework: concepts, structure, and extensibility. Statistics and Computing, 10:325–337.

© 2020 Mera JR et al. *JAMA Network Open.*
